# Adding effect sizes to a systematic review on interventions for promoting physical activity among European teenagers

**DOI:** 10.1186/1479-5868-7-29

**Published:** 2010-04-16

**Authors:** Rik Crutzen

**Affiliations:** 1 Maastricht University/CAPHRI, Maastricht, The Netherlands

## Abstract

This commentary adds effect sizes to the recently published systematic review by De Meester and colleagues and provides a more detailed insight into the effectiveness of interventions to promote physical activity among European teenagers. The main findings based on this evidence were: (1) school-based interventions generally lead to short term improvement in physical activity levels, but there were large differences between interventions with regard to effect sizes; (2) a multi-component approach (including environmental components) generally resulted in larger effect sizes, thereby providing evidence for the assumption that a multi-component approach should produce synergistic results; and (3) if an intervention aimed to affect more health behaviours besides physical activity, then the intervention appeared to be less effective in favour of physical activity.

## Commentary

The recently published systematic review by De Meester and colleagues [1] provides an excellent summary of the effectiveness of interventions to promote physical activity among European teenagers. The authors, however, based their conclusions merely on *p-*values while effect sizes are more appropriate to derive conclusions with regard to differences in the effectiveness of interventions. Therefore, in addition to the highly appreciated work done by De Meester and colleagues, I added effect sizes and provided an even more detailed insight into the effectiveness of interventions to promote physical activity among European teenagers.

Traditionally, null hypothesis-testing resulted in reporting of *p*-values as the key result of studies in behavioural sciences. Nowadays, however, reporting of effect sizes is deemed increasingly important [2]. Volker [3] argues on why to report effect sizes. In short, *p*-values are not particularly informative to determine whether a statistically significant effect is meaningful and substantive. They are merely "a conditional probability indicative of the probability of a result at least as extreme as the obtained difference assuming that the null hypothesis is true." [3] Therefore, effect sizes (i.e, Cohen's *d*) are reported in this commentary to gain a more detailed insight into the meaningfulness and substantiveness of the results of the studies included by De Meester and colleagues [1]. As correctly argued by De Meester and colleagues [1], performing a meta-analysis is problematic because of the heterogeneity of the outcome measures of the included studies. Nevertheless, effect sizes still provide the opportunity to quantify the intervention outcomes instead of having only a conditional probability. It needs to be stressed, however, that an effect size on its own is not equal to the public health impact of an intervention. According to the RE-AIM model, the public health impact of an intervention can only be evaluated by the assessment of five dimensions: Reach, Efficacy, Adoption, Implementation and Maintenance (hence the acronym RE-AIM) [4]. Thus, the public health impact of an intervention that has a small effect, but reaches a large group of people, can still be high. Furthermore, it is important, when looking at the potential of interventions, not to lose sight of the quality assessment of the studies (irrespective of the intervention outcomes).

Table 1 gives an overview of the effect sizes of intervention outcomes. Effect sizes were calculated by using the formulas described by Lipsey and Wilson [5]. As recommended by Morris [6], effect sizes were based on the pooled pre- and post-test standard deviation to obtain a more precise effect size estimate. Odds ratios were converted into Cohen's *d *(as described by Chinn [7]). Three studies did not report sufficient information to calculate effect sizes; one of the authors of these studies was able to provide the required information. In line with Cohen's classification [8,9], effect sizes were divided into five levels: trivial (Cohen's *d *≤ .2), small (> .2), moderate (> .5), large (> .8), and very large (> 1.3).

**Table 1:** Effect sizes of intervention outcomes

Study/country	E^1^	M^2^	Q^3^	Outcome measures	Cohen's	*d*^4^
***School setting***						
Haerens et al. (2007) [12], Belgium	N	N	3	Total PA level (min/day)	-0.01	
				School-related PA (min/day)	0.14	
				Leisure time sport (min/day)	-0.09	
				Leisure time active transportation (min/day)	0.05	
Verstraete et al. (2006) [13], Belgium	Y	N	2	Low intensity PA (% time spent during morning recess)	-0.30	*
				Moderate intensity PA (% time spent during morning recess)	0.53	**
				Vigorous intensity PA (% time spent during morning recess)	-0.12	
				Moderate to vigorous PA (% time spent during morning recess)	0.35	*
				Low intensity PA (% time spent during lunch break)	-1.06	***
				Moderate intensity PA (% time spent during lunch break)	0.89	***
				Vigorous intensity PA (% time spent during lunch break)	0.54	**
				Moderate to vigorous PA (% time spent during lunch break)	1.00	***
Hill et al. (2007) [11], UK	N	N	2	Exercise sessions min. 30 min/week without PE (leaflet only)	0.18	
				Exercise sessions min. 30 min/week without PE (leaflet plus quiz)	0.45	*
				Exercise sessions min. 30 min/week without PE (leaflet + implementation intention prompt)	0.32	*
Tsorbatzoudis (2005) [14], Greece	N	N	2	End of intervention: exercise habits (score)	0.59	**
Lubans and Sylva (2006) [15], UK	N	N	1	End of intervention: moderate to vigorous PA (min/week) of 20 min. or longer	0.65	*
Murphy et al. (2006) [16], Ireland	Y	N	1		^x^	
Lindberg et al. (2006) [17], Sweden	N	Y	1		^x^	
Chatzisarantis and Hagger (2005) [18], UK	N	N	1	Frequency of mild, moderate and vigorous PA during leisure time in the last 5 weeks	0.08	
Digelidis et al. (2003) [19], Greece	Y	Y	1	End of intervention: frequency of regular exercise in the previous month	-0.04	
***School setting with involvement of family***						
Harrison et al. (2006) [20], Ireland	Y	N	3	Moderate to vigorous PA: principal PA + intensity (30 min blocks/day)	2.07	****
Haerens et al. (2007) [21], Belgium	Y	N	2	Total PA level (min/day)	0.12	
				School-related PA (min/day)	0.39	*
				Leisure time sport (min/day)	-0.02	
				Leisure time active transportation (min/day)	0.07	
				Subsample accelerometer data: PA of light intensity (min/day)	0.53	**
				Subsample accelerometer data: PA of moderate to vigorous intensity (min/day)	0.50	**
Christodoulos et al. (2006) [10], Greece	Y	N	2	Organised moderate to vigorous PA (h/week)	3.53	****
				Total moderate to vigorous PA (h/week)	2.79	****
***School setting with involvement of family and community***						
Simon et al. (2006) [22], France	Y	N	2	Leisure supervised PA (%)	0.56	**
Jurg et al. (2006) [23], The Netherlands	Y	N	1	Total PA score (min/day at least moderately active)	0.11	
				Meeting the guidelines: 60 min/day of moderate PA (%)	0.27	*
Moon et al. (1999) [24], UK	Y	Y	1	Taking part in sports at school (not PE) once or more a week (%)	-0.02	
***Community with involvement of schools***						
Baxter et al. (1997) [25], UK	Y	Y	2	Students that exercise 3 or more times weekly (%)	0.40	*
***Primary care***						
Ortega-Sanchez et al. (2004) [26], Spain	N	N	1	6 months after 1st session: duration (min/week)	0.28	*
				6 months after 1st session: frequency (days/week)	0.17	
				6 months after 1st session: intensity in points (mild = 1, moderate = 2, vigorous = 3)	0.32	*
				12 months after 1st session: duration (min/week)	0.37	*
				12 months after 1st session: frequency (days/week)	0.25	*
				12 months after 1st session: intensity in points (mild = 1, moderate = 2, vigorous = 3)	0.44	*
Walker et al. (1999) [27], UK	N	Y	1	Teenagers who reported positive behaviour change (% 3 months after intervention)	0.14	
Kelleher et al. (1999) [28], Ireland	N	Y	1	Exercise (times/week) by 12-15 years old	< 0.01	
***Individual***						
Woods et al. (2002) [29], Scotland	N	N	2	Membership of the Sport and Recreation Service at the university (%)	0.61	**

^1^Intervention includes environmental components: Y(es)/N(o)

^2^Intervention aims to affect more health behaviours besides physical activity: Y(es)/N(o)

^3^Global rating quality assessment: three-grade scale (3 = strong; 2 = moderate; 1 = weak)

^4^Classification of effect sizes: trivial (Cohen's d ≤ .2); * small (Cohen's d > .2); ** moderate (Cohen's d > .5); *** large (Cohen's d > .8); **** very large (Cohen's d > 1.3)

^x^Impossible to calculate effect size based on reported results

The effects sizes in Table 1 clearly demonstrate the differences between effect sizes and *p*-values. For example, the *p*-value of the difference between the intervention group and the control group in the study by Christodoulos and colleagues [10], with regard to total moderate to vigorous physical activity, did not reach significance. The effect size for this outcome measure, however, was very large (*d *= 2.79). This demonstrates the potential of this intervention with regard to behaviour change, apart from the small sample size in the specific study. Another example, the study of Hill and colleagues [11], shows that although the conditions in this study did not differ significantly, the effect sizes indicate a clear difference between the impact of these interventions (ranging from 0.18 to 0.45).

Taking into account the effect sizes reported in Table 1, the conclusion of De Meester and colleagues [1] that school-based interventions generally lead to short term improvement in physical activity levels still holds. There were, however, large differences between interventions with regard to effect sizes. These differences should be taken into account when judging the potential of intervention solutions. Therefore, the evidence with regard to involvement of family is inconclusive and recommendations regarding family involvement should be interpreted with caution as they are premature. In contrast to De Meester and colleagues [1], the evidence provided by effect sizes is not inconclusive with regard to a multi-component approach. Interventions that included environmental components (as identified in the published review) generally resulted in larger effect sizes. This provides evidence for the assumption that a multi-component approach should produce synergistic results. With regard to interventions aimed at multiple behaviours, it can be concluded that these interventions resulted in smaller effects with regard to physical activity. There appeared to be no differences in effect sizes related to quality assessment of the studies (as assessed in the published review). Nevertheless, when homogeneous outcome measures are available for future studies, meta-analyses are needed to fully warrant strong conclusions with regard to potential moderators of effect sizes (e.g., methodological quality).

## Conclusions

Based on the evidence identified by the review of De Meester and colleagues [1] and the effect sizes reported in this commentary, a detailed insight into the effectiveness of interventions to promote physical activity among European teenagers is provided. In summary, the main findings based on this evidence:

(1) School-based interventions generally lead to short term improvement in physical activity levels, but there were large differences between interventions with regard to effect sizes.

(2) A multi-component approach (including environmental components) generally resulted in larger effect sizes, thereby providing evidence for the assumption that a multi-component approach should produce synergistic results.

(3) If an intervention aimed to affect more health behaviours besides physical activity, then the intervention appeared to be less effective in favour of physical activity.

## Competing interests

The author declares that they have no competing interests.

## Authors' contributions

RC synthesized the data and calculated the effect sizes, interpreted the findings, and wrote the manuscript.
